# Leveraging large language models for peptide antibiotic design

**DOI:** 10.1016/j.xcrp.2024.102359

**Published:** 2024-12-31

**Authors:** Changge Guan, Fabiano C. Fernandes, Octavio L. Franco, Cesar de la Fuente-Nunez

**Affiliations:** 1 Machine Biology Group, Departments of Psychiatry and Microbiology, Institute for Biomedical Informatics, Institute for Translational Medicine and Therapeutics, Perelman School of Medicine, University of Pennsylvania, Philadelphia, PA, USA; 2 Departments of Bioengineering and Chemical and Biomolecular Engineering, School of Engineering and Applied Science, University of Pennsylvania, Philadelphia, PA, USA; 3 Department of Chemistry, School of Arts and Sciences, University of Pennsylvania, Philadelphia, PA, USA; 4 Penn Institute for Computational Science, University of Pennsylvania, Philadelphia, PA, USA; 5 Centro de Análises Proteômicas e Bioquímicas, Pós-Graduação em Ciências Genômicas e Biotecnologia, Universidade Católica de Brasília, Brasília, Brazil; 6 Departamento de Ciência da Computação, Instituto Federal de Brasília, Campus Taguatinga, Brasília, Brazil; 7 S-Inova Biotech, Programa de Pós-Graduação em Biotecnologia, Universidade Católica Dom Bosco, Campo Grande, Brazil; 8 These authors contributed equally

## Abstract

Large language models (LLMs) have significantly impacted various domains of our society, including recent applications in complex fields such as biology and chemistry. These models, built on sophisticated neural network architectures and trained on extensive datasets, are powerful tools for designing, optimizing, and generating molecules. This review explores the role of LLMs in discovering and designing antibiotics, focusing on peptide molecules. We highlight advancements in drug design and outline the challenges of applying LLMs in these areas.

## INTRODUCTION

Large language models (LLMs) are pushing the boundaries of innovation across various fields Powered by advancements in artificial intelligence (AI), neural network architectures, and hardware such as graphics processing units (GPUs), LLMs have initiated a new era of “AI for science.” These models can support researchers in generating hypotheses, designing experiments, collecting databases, and analyzing data, thereby enhancing and accelerating scientific research.^1–4^ Their applications span chemistry,^5^ biology,^6^ medicine,^7^ materials science,^8^ and beyond.

LLMs, a class of AI algorithms, utilize deep learning and extensive datasets to perform natural language processing (NLP) tasks, effectively generating human-like text. These tasks include text generation, language translation, summarization, question answering, and sentiment analysis.^9^ Unlike early language models based on statistical methods and n-gram models,^10^ LLMs can comprehend associative relationships within sentences and texts within extensive databases. These databases often contain parameters ranging from tens of millions to billions.^11^

Pre-trained language models (PLMs), such as those using multi-head attention mechanisms like the transformer,^12^ have become increasingly popular. These models, which can contain up to hundreds of billions of parameters, excel in “few-shot” or “zero-shot” learning—abilities that allow them to perform tasks with minimal task-specific data, unlike smaller-scale models like the bidirectional encoder representations from transformers (BERT).^11^

LLMs have also achieved significant advancements in specialized applications. For example, chat generative pre-trained transformer (ChatGPT), a generative AI chatbot, leverages the decoder from the transformer^12^ model and multimodal technologies, forming the basis for GPT-3.5 and GPT-4.^13,14^ ChatGPT has demonstrated competencies comparable to human performance in cognitive tasks, including academic writing,^15^ and has significantly impacted fields such as chemistry,^16^ biology,^17^ and medicine^7^ by enhancing functions like chemical representations in SMILES format.^16^

Furthermore, LLMs have achieved significant advances in protein structure prediction. Models such as RNG2,^18^ for instance, accurately predict protein structures, and ESM-1 has been analyzed over 617 million protein sequences.^19^ These models use structural data from proteins to design novel protein structures tailored for specific functions,^20–22^ enhancing fields like protein engineering, biomolecular design, and drug discovery.^23,24^

Peptide design is an area of research where LLMs can have a significant impact. For example, antimicrobial peptides (AMPs) constitute a potential alternative to conventional antibiotics^2,3^ and computational approaches have been recently developed for their design and discovery.^4,25^

This review focuses on the role of LLMs in AMP and antibiotic design, highlighting their potential in drug discovery.

## HISTORY OF LLMs

**Figure 1.**
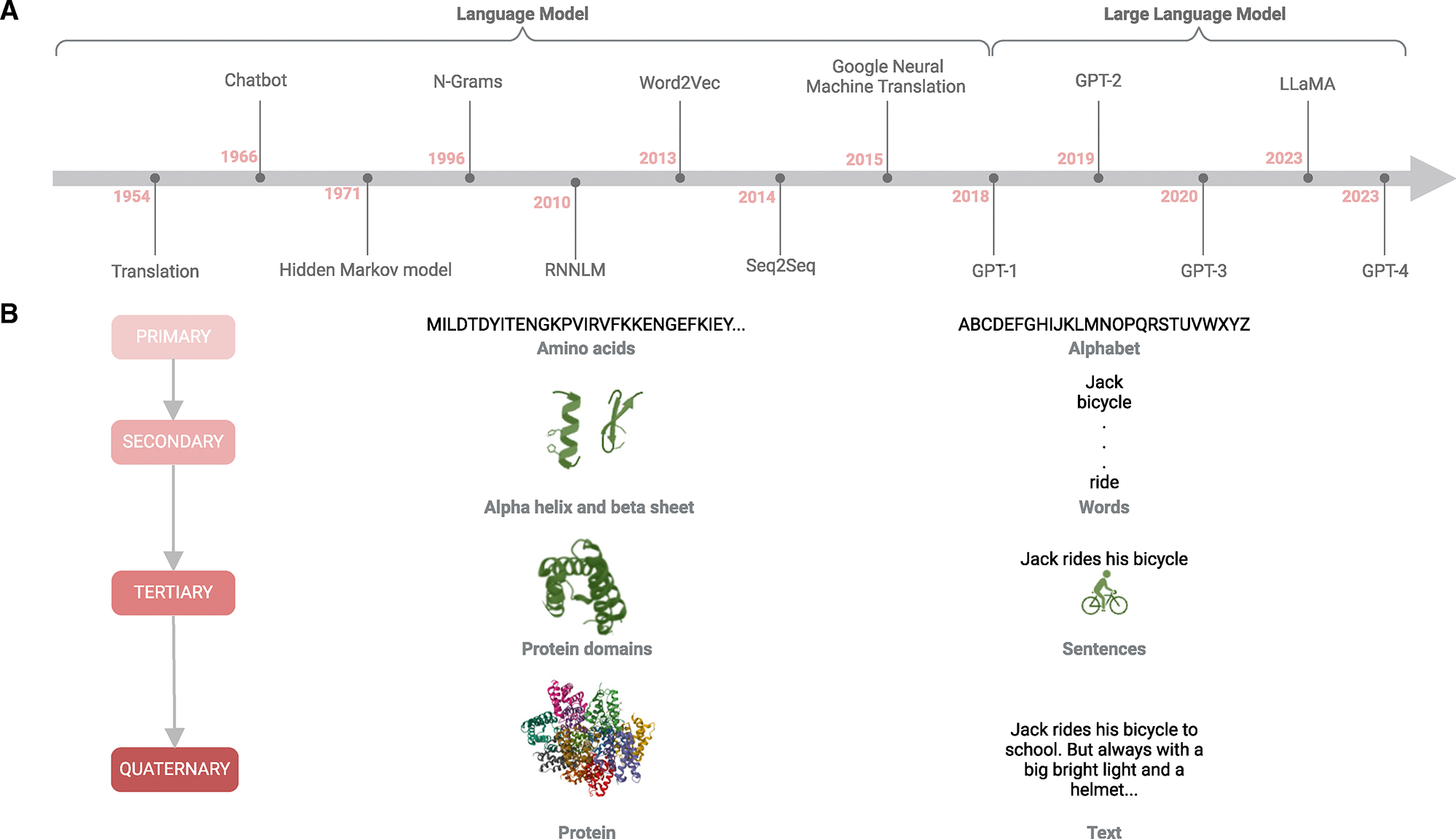
Timeline of advances in language models and similarities between proteins and language (A) This timeline illustrates the evolution of language models alongside advancements in AI, beginning with early translation models (e.g., Russian to English) and advancing to sophisticated large language models like GPT and LLaMA. Each language model is represented within a circle, clearly labeled with the model’s name and year of development. (B) Protein sequences (primary structure) are depicted as 20 characters, each representing an amino acid. These amino acids fold into three-dimensional secondary structures, such as a helices and b sheets, similar to how words form meaningful sentences. These tertiary structures are responsible for specific biological functions. Additionally, protein domains combine to create larger quaternary complexes, much like sentences combine to form a coherent text.

LLMs have evolved significantly since their inception in the 1950s and 1960s^26^ (Figure 1A). Early models relied on handcrafted linguistic rules, followed by probabilistic methods in the 1970s and 1990s^27^ (Figure 1A). However, these models struggled to handle the complexities of language, particularly its ambiguities, and lacked robust generalization across the unseen text. Building on these foundations, the basic local alignment search tool (BLAST) was introduced as a heuristic algorithm for comparing biological sequences,^28^ while hidden Markov models (HMMs), adapted from NLP, were applied to identify gene structures, conserved regions, and domains, revealing evolutionary relationships.^29^ This work opened the door for NLP applications in biology, especially within bioinformatics and computational biology.

A significant leap occurred in the mid-2010s with the advent of neural network models. This era saw the development of deep neural network language models (Figure 1A), including those based on recurrent neural networks (RNNs),^30^ extended short-term memory networks (long short-term memory [LSTM]),^31^ and convolutional neural networks (CNNs).^32^ The first deep neural network model was the RNN LM, introduced in 2010.^33^ RNNs introduced the concept of recurrence, where the output from a previous time step is fed to the current step. This allows the hidden layer of an RNN to receive input from the current word in the sequence and the secret state from the previous time step and gives the RNN memory to capture dependencies between words. However, there is a vanishing gradient problem in RNNs, where the gradients of the loss function become too small to update the weights properly during backpropagation, which makes it difficult for RNNs to learn long-range dependencies effectively.^34^ To address the vanishing gradient problem in RNNs, gating mechanisms—input gate, forget gate, and output gate—that help decide which information to keep were introduced. This architecture allows LSTMs to retain essential details over longer sequences. However, LSTM is computationally more expensive due to its complex gate mechanisms. Therefore, LSTM still has limitations in training efficiency and struggles with very long sequences, especially when parallelization is desired for scaling.^35^ CNNs were adapted for NLP tasks by using convolutions over sequences of words and filter slides over word embeddings to detect patterns (like n-grams). CNNs can capture local features effectively and in parallel and are faster than RNNs and LSTMs because they do not depend on previous time steps to process the current word. However, CNNs struggle to capture global context, making them less suitable for tasks requiring an understanding of the entire sequence.^36^ While these deep learning methods have advanced bioinformatics and computational biology, RNNs are designed explicitly for sequential data, making them ideal for analyzing biological sequences such as proteins and RNA, time-series data, and gene regulatory networks.^37^ LSTM networks, a type of RNN, are particularly effective at managing long-term dependencies in sequences and are applied to tasks like protein structure prediction, gene expression analysis, and drug-target interaction prediction.^38^ CNNs, in contrast, are often used for analyzing spatial or structured data, which makes them powerful tools for image-based biological tasks and sequence analysis, including medical imaging, protein structure prediction, and genomics.^39^

Subsequent innovations included the introduction of Word2Vec models and sequence-to-sequence (Seq2Seq) models in 2013 and 2014.^40,41^ The same period saw the introduction of attention mechanisms, a significant milestone that significantly improved the performance of Seq2Seq models.^42^ These advancements culminated in the development of the Google neural machine translation (GNMT)^43^ system in 2015, a large-scale language model leveraging LSTM with 278 million parameters^44^ (Figure 1A). Word2Vec introduced word embeddings mapping words into continuous vector spaces, with similar words appearing close together in latent space. It has two main approaches: CBOW (continuous bag of words), predicting a word based on its context, and Skip-gram, predicting the context based on a word. Seq2Seq architecture, typically using LSTM, maps an input sequence (such as a sentence) processed by the encoder to an output sequence generated by the decoder (like a translated sentence). However, Seq2Seq models rely heavily on LSTM and thus inherit their limitations, particularly regarding handling long sequences and inefficient parallelization during training. In GNMT, the attention mechanism, weighing the importance of different words in the input sequence when making predictions, was applied to LSTM to improve translation accuracy by focusing on the most relevant parts of the input sentence. Although the attention mechanism made GNMT better suited for long sentences and sequences with complex dependencies, it still faced challenges with scalability and speed due to its reliance on LSTMs.

A pivotal advancement in language models occurred in 2017 with the introduction of the transformer model. This model features a self-attention mechanism that effectively eliminates sequence-length limitations, enabling it to handle long text sequences better.^12^ The transformer architecture, which utilizes parallelization and distributed processing, has been applied across diverse information domains and computational tasks.^12^

Adopting transformer models marked the beginning of the era for LLMs. The first GPT-1, developed by OpenAI, featured over one billion parameters and was trained on an extensive text corpus using the GPT framework^13^ (Figure 1A). This model, along with other PLMs such as BERT and subsequent versions of GPT, has excelled in capturing contextual information. These PLMs have been fine-tuned for specific downstream tasks, leading to highly successful outcomes across various applications in NLP.

As the scale of parameters in PLMs increases, researchers have observed that larger models tend to demonstrate enhanced performance on downstream tasks. This phenomenon is attributed to what is known as emergent abilities,^45^ increasing the model’s size, using consistent architectures, and applying varied pre-training tasks can lead to unique capabilities.

For instance, GPT-3, with its vast parameter count, excels at few-shot learning tasks, showing significant improvement over its predecessor, GPT-2, which struggles with these challenges. These improvements have led to categorizing such models as large-sized PLMs with emergent abilities, typically referring to transformer-based language models that house hundreds of billions of parameters.^45^ Pursuing these emergent abilities has spurred the development of even larger models, such as GPT-3.5,^13^ GPT-4,^14^ and LLaMA^46^ (Figure 1A).

In summary, early models (n-grams, rule based) were limited by their inability to capture complex dependencies and required handcrafted rules. Deep learning architectures like RNNs and LSTM introduced memory and improved the handling of sequential dependencies but struggled with vanishing gradients and inefficiency in long texts. CNNs enabled parallel processing but could not capture the global context. Seq2Seq and attention mechanisms advanced sequence handling but still depended on LSTM. Transformers overcame these limitations with parallel processing, scalability, and the ability to capture long-range dependencies efficiently, revolutionizing modern NLP.

Biological sequences such as DNA, RNA, and proteins can be conceptualized as code or language,^47^ complete with unique characters, syntax, and semantics (Figure 1B). For example, amino acid sequences, the building blocks of proteins, comprise 20 standard amino acids. These sequences fold into secondary structures such as α helices and β sheets, which then arrange into complex tertiary structures. This structural organization is analogous to how letters form words and words form sentences in human language, each conveying specific meanings within their respective languages.

Protein structures essential for specific biological functions can be likened to sentences constructing cohesive texts.^48^ Representing these biological sequences as vectors within vector spaces is crucial for effectively leveraging machine learning in biological research. Many researchers have applied natural language theories, machine learning, and other analytical methods to develop robust biological language models. LLMs are promising, as they utilize higher-dimensional vectors to decode and represent this complex biological language effectively.

## THE TRANSFORMER ARCHITECTURE AND ITS VARIANTS

**Figure 2.**
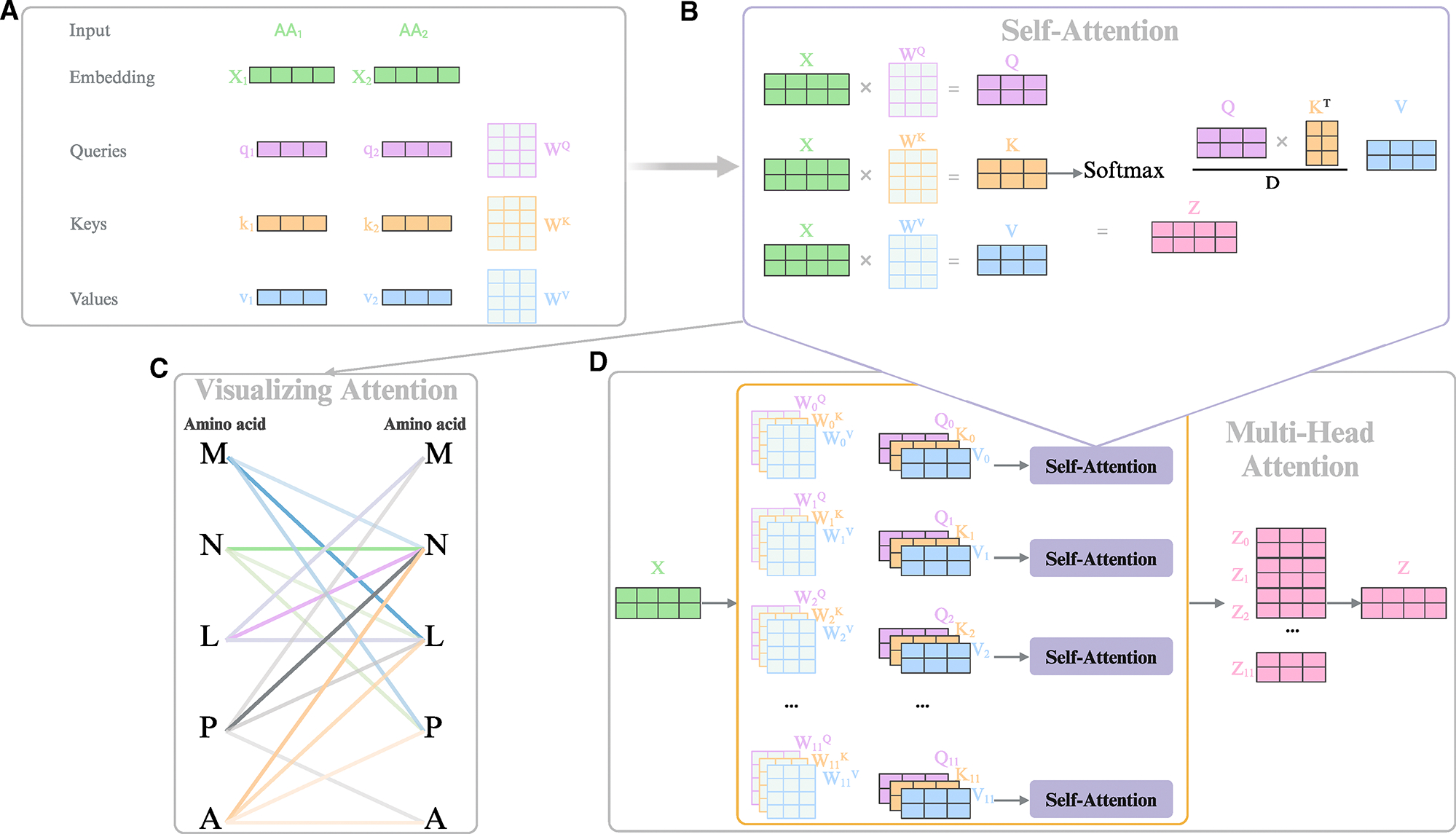
The self-attention and multi-head self-attention mechanism (A) Multiplying *x*_*1*_ by the *W* weight matrix produces the “query,” “key,” and “value” projection vectors for each word in the input sentence. (B) Attention scores are calculated by taking the dot product of the query and key matrices. These scores are then normalized using a softmax function, and the weighted sum of the value matrix is computed based on the attention weights to produce the output of the self-attention layer. (C) Attention scores representing mutual attention between each pair of amino acids are visualized, with each color indicating a specific amino acid type. Darker colors signify a higher level of attention between amino acids. (D) In multi-head self-attention, multiple query and key matrices compute several weighted sums of the value matrices. Outputs from these self-attention layers are then concatenated and linearly transformed using a learned weight matrix to generate the final output.

Compared to CNN models, which primarily capture local dependencies via convolution kernels, transformer models can capture both global and local dependencies in a sequence. Additionally, unlike RNNs, which struggle with processing long sequences and are constrained to unidirectional processing, transformers can efficiently process bidirectionally and handle long sequences, eliminating the need for recurrence and convolutions. This capability is made possible by the self-attention mechanism (Figure 2), which enables the transformer model to process all elements of the input sequence simultaneously and focus on the most relevant aspects, capturing both global and local dependencies.^12^

For example, given an amino acid sequence, the model embeds each amino acid into a vector with positional information and derives query (Q), key (K), and value (V) vectors by transforming the embedding (Figures 2A and 2B). The self-attention mechanism can be described as mapping a Q vector and a set of K-V pairs into an output vector (Figure 2B). The dot product between the Q vector of “AA1” and the K vector of “AA2” generates a scalar (attention score) that indicates how much attention AA1 directs toward AA2. For a peptide sequence, the attention score reflects the attention between each pair of amino acids, helping to elucidate relationships within the sequence and identify important domains or motifs related to function (Figure 2C). Consequently, self-attention enables researchers to study peptide or protein sequences more effectively. This attention score then scales the corresponding V vector, preserving mutual attention information within the representation vector of each amino acid and allowing the model to focus on the most relevant amino acids (Figure 2B).

Transformer models employ a multi-head self-attention mechanism consisting of multiple attention heads that compute attention weights independently. This allows the model to attend to information from different representation subspaces at various positions. The outputs from each attention head are then concatenated and linearly transformed to produce the final output (Figure 2D).

**Figure 3.**
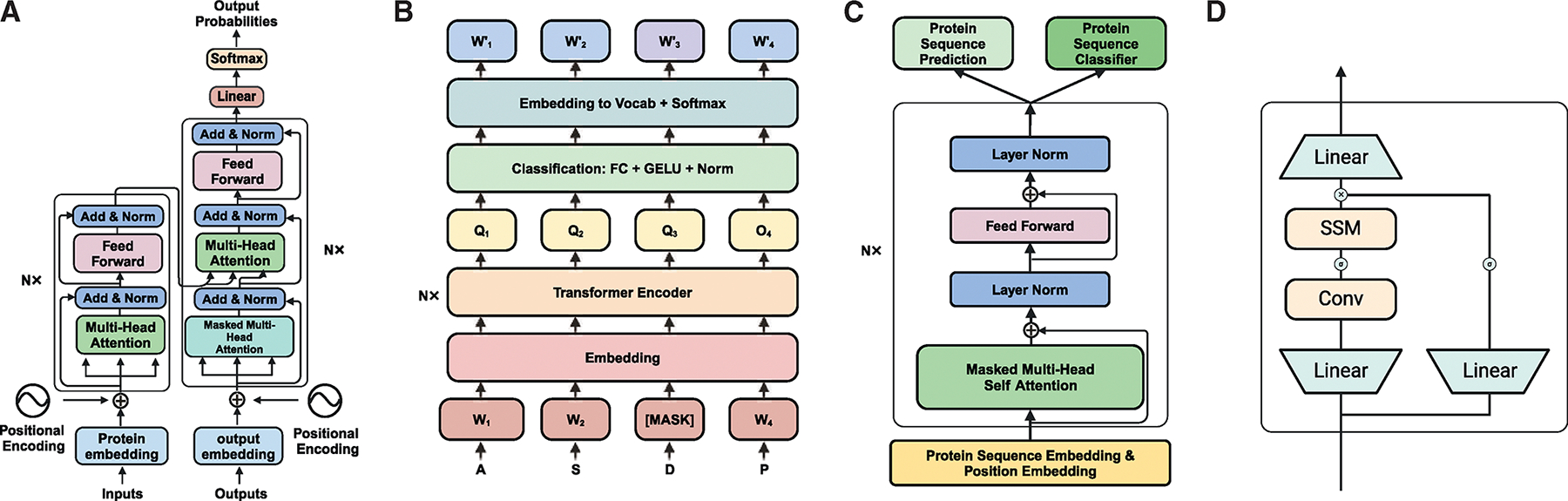
Transformer, BERT, GPT, and Mamba architectures (A) The transformer architecture includes an encoder for processing inputs and a decoder for generating outputs. (B) BERT architecture consists of self-attention layers. (C) The GPT architecture comprises masked self-attention layers. (D) The Mamba architecture is composed of conventional layers and a state-space model.

Based on the attention mechanism, several transformer architectures have been developed, mainly falling into three types: encoder architectures like BERT, decoder architectures like GPT, and encoder-decoder architectures like the original transformer^49^ model (Figure 3). The original transformer neural sequence transduction model, which includes both an encoder and decoder, was designed to handle Seq2Seq tasks (Figure 3A). The encoder processes the input sequence (such as a protein sequence) and generates a sequence of vectors representing the input. The decoder then generates the output sequence one token at a time based on the vectors from the encoder. The critical difference between the encoder and decoder lies in the attention mechanisms: the decoder uses masked multi-head attention, which restricts it to seeing only the tokens before the current token (forming an upper triangular matrix), while the encoder uses unmasked multi-head attention, allowing it to attend to the entire input sequence.

The BERT variant of the transformer was developed to obtain better sequence representations. BERT uses only the encoder part of the transformer architecture and unmasked multi-head attention,^50,51^ allowing it to capture context from both directions of a sequence simultaneously (Figure 3B). In contrast, the GPT variant was proposed for tasks such as sequence generation or text creation (Figure 3C). GPT^51^ is a unidirectional transformer architecture that utilizes a multi-head attention mechanism to attend to different parts of the input sequence simultaneously and weigh their importance.

There are several other transformer architecture variants based on the original three structures. For instance, the encoder-based model BigBird^52^ uses compound position-based sparse attention to handle long sequence inputs effectively. GPT-3^53^ incorporates alternating dense and locally banded sparse attention in its self-attention modules. The switch transformer,^54^ another encoder-based model, replaces traditional feedforward network layers with a mixture of expert layers, allowing it to increase the parameter count while maintaining constant floating-point operations, for example. Significantly, the Evoformer architecture in AlphaFold2 is specially designed to capture evolutionary conservation from multiple sequence alignments, enhancing the accuracy of protein structure prediction. Although these variants are primarily used in NLP, a new architecture called Mamba^55,56^ has been introduced, which features an RNN-like token mixer based on a state-space model (SSM), offering a challenge to the transformer architecture (Figure 3D). This model has been pre-trained on protein^57^ or gene sequences.

Several pre-trained tasks have been proposed to train transformer models, including masked language modeling (MLM), next sentence prediction (NSP), autoregressive language model (LM), sentence order prediction (SOP), span boundary objective (SBO), and replace token detection (RTD), among others.^58^ MLM, NSP, and LM are used in LLMs for biology and chemistry. Therefore, these tasks are detailed below, and their loss functions are listed in Table 1.

## MLM

In this task, certain input tokens are randomly replaced with a [MASK] token. The model is then trained to predict the original token that was replaced. This training strategy is consistently applied to BERT models and helps them learn semantic information, improving the model’s ability to represent sequences effectively.

## NSP

This task helps the model understand inter-sentence coherence. The model is trained to determine whether sentence B follows sentence A by using two consecutive segments from the same document as positive examples and the same two segments with their order swapped as negative examples. NSP is commonly used in BERT models to enhance sentence representation.

### Autoregressive language modeling

This task involves training the model to predict the next token in a sequence based on the preceding tokens. It is a classic probabilistic density estimation problem and is used for GPT models and generative tasks.

By using these tasks to pre-train LLMs on protein or nucleic acid sequences, LLMs can learn the underlying semantic information of these sequences, including functional insights. Consequently, these models can assist researchers in understanding biological sequences and uncovering the underlying codes and principles of life embedded within them.

## PROTEIN LLMs: APPLICATIONS OF PEPTIDE SEQUENCES

**Figure 4.**
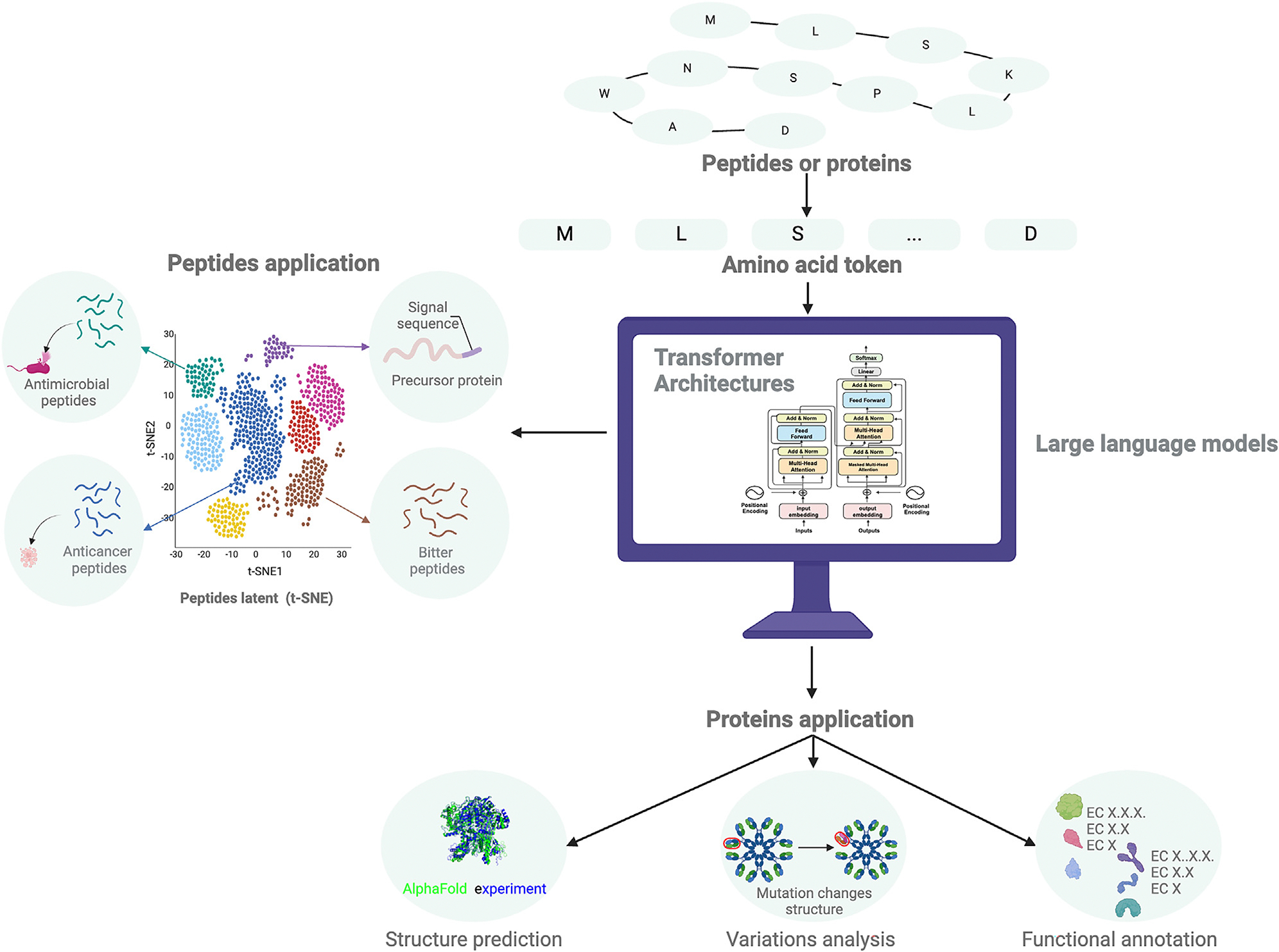
Applications of LLMs in protein and peptide design This figure illustrates using LLMs with a transformer architecture for designing peptides and proteins. Amino acid sequences are segmented per amino acid and input into LLMs (shown in the right box). The resulting representations of peptides, derived from LLMs, facilitate the classification of peptides based on different functions (left box). Additionally, LLMs are utilized for various protein-related tasks, including structure prediction, variations analysis, and functional annotation (bottom box).

LMs are not limited to processing natural language text but exhibit significant potential for analyzing amino and nucleic acid sequences. In recent decades, biologists have experimentally revealed vast amounts of protein and genetic data, fueling the development of biological LLMs. For example, LLMs have been applied for amino acid representation, peptide property prediction, protein structure prediction, sequence function annotation, and variant analysis (Figure 4).

Amino acids are the fundamental building blocks of peptides and proteins, playing a critical role in their structure and function. By creating a numerical vocabulary to encode each amino acid and utilizing self-supervised pre-training tasks, LLMs can learn to represent amino acid sequences effectively.^59^ These representations can capture the complex interactions between amino acids, facilitating the identification of patterns and functional regions within sequences.^60^ Demonstrably superior to traditional methods, these representations enhance the functional analysis of proteins.^6^ With pre-training on extensive protein databases, LLMs develop detailed amino acid representations illuminating the intricate relationships between sequence, structure, and function,^22^ making them increasingly valuable for protein research.

LLMs leverage sequence patterns and positions to predict secondary and tertiary structures, foldability, and other protein properties.^18,19,21^ They can correlate protein sequences with their functions. As a result, LLMs can be used for functional annotation of unknown proteins,^61^ analysis of sequence variations, and prediction of their effects on protein structure and function^62^ (Figure 4). Functional annotation also helps reveal connections between genetic variation, diseases, and protein evolution. Numerous protein LLMs have been developed, each performing differently based on parameters such as architecture, layers, attention heads, and training datasets. Users must select models based on their specific tasks (Table 2). While we will not delve deeply into protein LLM applications, we focus on peptides and AMPs, which rely on these protein LLMs.

**Figure 5.**
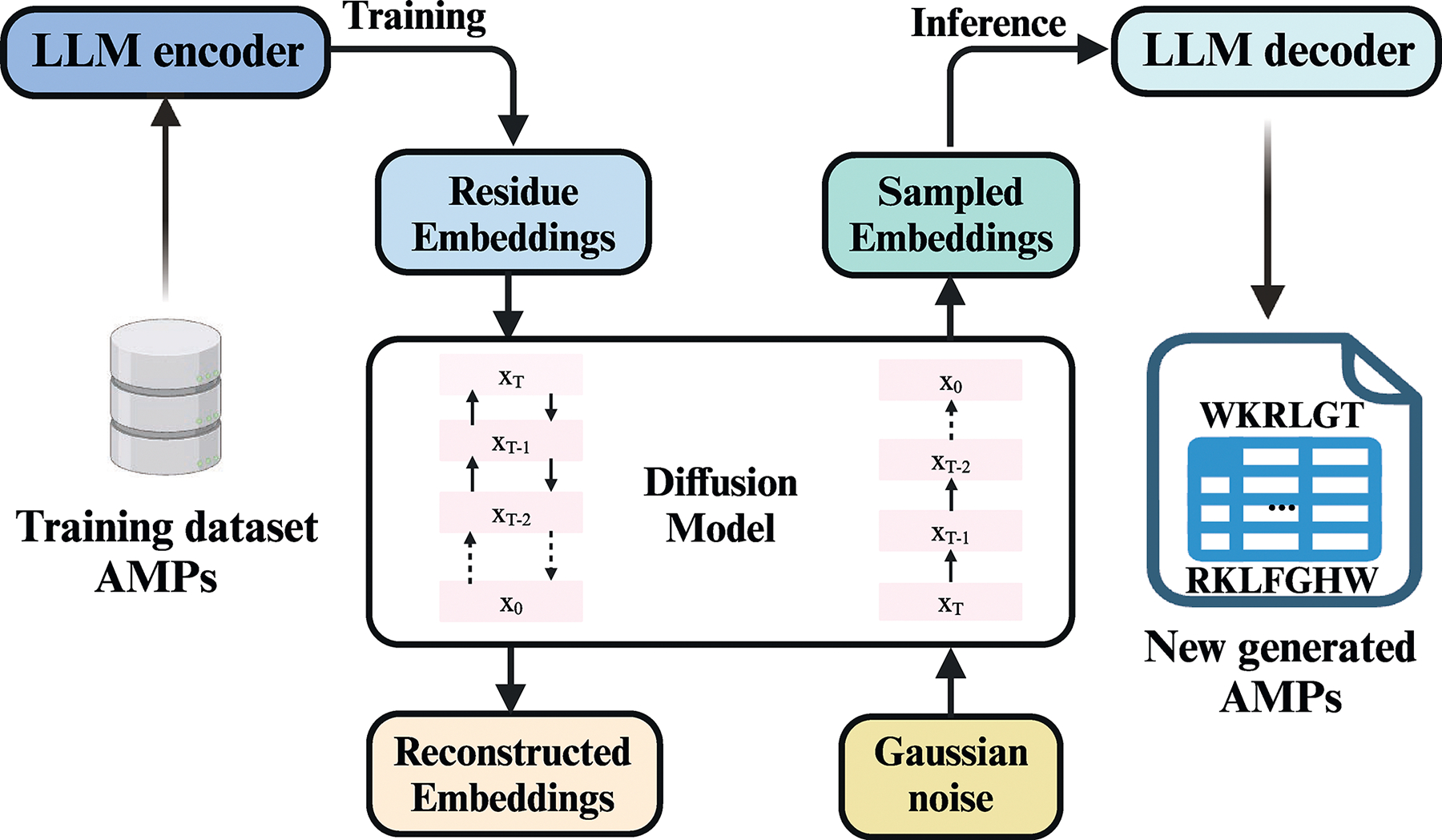
Training and fine-tuning an LLM for AMP classification tool development This figure illustrates the integration of LLMs and diffusion models in developing tools for AMP classification. LLMs are employed to generate new AMP sequences; however, they often exhibit limited diversity in the latent space representations of non-natural AMPs. To overcome this, diffusion models introduce variations in sequences and structures. These variations are then reconstructed within the LLM embeddings, enabling the generation of novel, functionally potent molecules. This synergy between LLMs and diffusion models enhances the potential for discovering AMPs with targeted functions. Figure created with BioRender.com.

Peptides, short chains of 2–50 amino acids linked by peptide bonds, form when the carboxyl group of one amino acid reacts with the amino group of another amino acid, releasing a water molecule.^63^ Peptides play various roles in biological systems, including antimicrobial activity.^64^ While computational methods exist to identify functional properties of peptides, their efficacy remains limited. LLMs enhance the speed of identifying bioactive peptides—such as signal peptides,^65–68^ anticancer peptides (ACPs),^69,70^ bitter peptides,^71^ and AMPs^25,64^—by utilizing vector representations of peptide sequences learned through LLMs (Figure 5). Moreover, models based on protein LLMs were developed to identify antihypertensive peptide,^72^ allergenic peptides,^73^ toxic peptides,^74^ antigenic peptides,^75^ major histocompatibility complex (MHC) binding peptides,^76,77^ antioxidant peptides,^78^ or human leukocyte antigen (HLA) class I binding peptides.^79^These models assist in identifying crucial motifs, domains, and potential binding sites for peptide functionality.^60^

Beyond bioactivity prediction, LLMs have significantly advanced peptide design and optimization. Peptides targeting proteins or other receptors can now be designed using LLMs.^80–84^ For example, PepPrCLIP^81^ focuses on *de novo* peptide motif generation without relying on structural data, using only sequence information. Cut&CLIP^82^ introduces a contrastive learning approach to design peptide-based protein degraders, which induce the degradation of specific target proteins. Peptide design with direct preference optimization (DPO)^84^ employs multi-objective optimization, balancing factors such as binding affinity, stability, and specificity. PepMLM, using a span-masked language model conditioned on target sequences, was developed for therapeutic peptide binder generation. Data-centric models^83^ emphasize the importance of large-scale, high-quality datasets when training generative language models for peptide design. Together, these models showcase various approaches to peptide binder design using protein language models (pLMs).

Additionally, due to the significant impact of structure on function, LLMs are expected to predict peptide structures, such as contact maps,^85^ aiding researchers in designing novel peptides^18^ and predicting peptide-protein interactions. For example, SaLT&PepPr^86^ focuses on designing peptides that facilitate protein degradation by predicting interaction interfaces from protein sequences for generating peptidic binding motifs. E2EPep combines LLMs with a cross-attention mechanism to predict binding residues between proteins and peptides, while TULIP^87^ was developed to predict interactions between peptides and T cell receptors (TCRs), specifically targeting unseen epitopes. PepNN^88^ uses a deep-attention-based neural network to identify peptide binding sites on proteins, and PepCNN^89^ leverages CNNs to predict peptide binding residues by incorporating sequence, structure, and language model features. These models represent advances in protein-peptide interactions driven by LLMs.

Furthermore, LLMs have made significant advances in peptide sequencing. PowerNovo,^90^ an innovative approach for *de novo* peptide sequencing, uses tandem mass spectrometry (MS/MS) data and an ensemble of transformer and BERT models, marking a significant advancement in this field.

## THE APPLICATION OF PROTEIN LLMs IN ANTIBIOTIC DISCOVERY

One of the essential urgent areas of research today is the need to develop new antibiotics to counter antimicrobial resistance.^91^ The integration of LLMs is expected to facilitate the search for new antibiotics. LLMs are being introduced into drug discovery for virtual screening, new drug compound identification, and antibiotic discovery.^92,93^ Specifically in antibiotic research, LLMs^94^ facilitate the exploration of diverse chemical spaces,^95^ predict compound interactions,^96^ and optimize antibiotic candidates.^97^ By processing extensive data on chemical compounds and their interactions with pathogens, LLMs can accelerate the identification of potential drug candidates with high binding affinities to target bacteria or viruses, expediting the drug development process from laboratory to clinic.^98^ Moreover, LLMs assist in discovering natural antibiotic sources from soil bacteria or fungi, guiding researchers through genetic data and chemical structures.^25^ LLMs can also help predict antibiotic resistance in pathogens^99–101^ by analyzing bacterial genetic sequences to detect potential mutations or resistance mechanisms,^102^ thereby informing the selection of the most effective antibiotic candidates for further development.

LLMs used in AMP research are primarily based on protein-focused LLMs pre-trained on protein sequences, though some natural language models have also been employed.

These models have demonstrated an ability to learn effective representation vectors to distinguish between different protein sequences.^6^ However, no conclusive evidence exists to identify which protein LLM best represents AMPs for AMP-related tasks. A recent study compared the ESM2 models of varying parameters for AMP classification but did not include comparisons of the 15B and 3B ESM2 models.^103^ As news continues to emerge for protein-related tasks (Table 2), it is increasingly urgent to determine which model is optimal for representing AMPs. Notably, as far as we know, no LLMs have been pre-trained specifically on short peptides (under 100 residues), highlighting a need to compare protein LLMs’ representation performance on short peptides versus longer protein sequences.

For AMP property prediction tasks, there are two ways to develop models by utilizing different protein LLMs or NLP LLMs: fine-tuning protein LLMs or NLP LLMs, or inputting AMP representation vectors derived from protein LLMs or NLP LLMs to another shadow model (Table 3). For the first approach, several models have been developed for the AMP identification task, including AMP BERT,^104^ iAMP-bert,^105^ AMP-ProteinBERT,^106^ Bert-Protein,^64^ and cAMPs_pred^25^; the main difference among these models is that they use different protein LLMs or NLP LLMs. Additionally, TransImbAMP^107^ employs the foundation LLM Tape, fine-tuned for predicting AMPs and their functional activities against different strains. PeptideBERT,^108^ on the other hand, utilizes the ProtBERT transformer model to predict key peptide characteristics such as hemolysis, solubility, and resistance to fouling. KT-AMP^109^ is based on the ProtT5 foundation LLM, fine-tuned to predict AMPs and additional functions, including antiviral activities. Notably, the NLP-based LLM GPT-3^110^ has also been fine-tuned to predict AMP antimicrobial activity and hemolysis.

In the second approach, feature vectors are fed into machine learning or deep learning models. For instance, LM_pred^111^ combines feature vectors extracted from the protein LLM ProtTrans with a CNN to predict AMPs. To further enhance the feature vector, iAMP-Attenpred^112^ incorporates CNNs, LSTM, and attention layers to predict AMPs more effectively. Additionally, PepNet^113^ processes feature vectors by inputting them into a new transformer block for AMP prediction.

Some models utilize multiple feature vectors derived from different protein LLMs. For example, AMP-Detector^114^ combines feature vectors from ProtTrans and ESM1b along with additional physicochemical features to predict the antimicrobial, antiviral, and antibacterial activities of AMPs. UniproLcad^115^ extracts feature vectors from ESM-2 and ProtBERT and then inputs these into a CNN and attention module for AMP prediction. SenseXAMP^116^ uses a cross-modal framework to merge feature vectors from ESM1b with protein descriptors for AMP prediction.

Overall, these LLM-based models can help expedite AMP discovery, potentially accelerating the identification of potential AMPs and significantly reducing experimental time. However, the accuracy of these models still requires experimental validation to confirm their predictions.

## USING LLM GENERATIVE MODELS AND GENERATIVE DIFFUSION MODELS FOR MOLECULAR DESIGN

Traditional molecular design approaches, such as X-ray crystallography, nuclear magnetic resonance (NMR), and molecular docking, predict interactions between small molecules and biological targets or use high-throughput screening to assess large compound libraries. These methods are constrained by human intuition and the need for a detailed mechanistic understanding of molecular interactions. The advent of machine learning models, including quantitative structure-activity relationships and random forest classifiers, automated the identification of these interactions. However, these models required extensive feature engineering and struggled to generalize to unseen data, limiting their ability to explore novel molecular spaces.

Recent developments in AI-based protein design^117,118^ have focused on generative models, such as variational autoencoders (VAEs), generative adversarial networks (GANs), diffusion probabilistic models, and pLMs.^119–121^ These models have demonstrated significant progress in generating novel and functional protein variants. However, VAEs and GANs primarily capture the distribution of the training data, making it challenging for them to generate sequences beyond that distribution or grasp semantic relationships. In contrast, LLMs have potential in designing novel proteins with unknown structures. By leveraging vast chemical and biological datasets, LLMs can learn generalizable representations of molecules, overcoming the limitations of feature engineering and narrow datasets. They can also understand the semantic connections between molecular structures and their biological functions.^122^ Additionally, LLMs excel in multi-objective optimization, balancing properties like activity, toxicity, and solubility—areas where traditional methods often fail.

For example, ProtGPT2, an LLM trained on protein sequences, can generate *de novo* protein sequences that follow the principles of natural proteins and explore unexplored regions of protein space.^123^ The model can also generate new AMP sequences by fine-tuning AMP datasets.^124^ Additionally, by introducing conditional constraints, ProGen can create proteins with desired properties, achieving fine-grained control over metrics like primary sequence similarity, secondary structure accuracy, and conformational energy.^48,125^ However, this model has not yet been applied to designing new AMPs.

The prefix-tuning method was introduced to enhance the design capabilities of ProtGPT2. PrefixProt^126^ utilizes prefix tuning to apply virtual tokens as control tags for specific protein properties.^46^ This method optimizes a small, continuous task-specific vector, known as the prefix, to precisely control protein generation without modifying other model parameters. Trained on datasets such as α helix structures, AMPs, and ACPs,^127^ PrefixProt effectively prompts pre-trained pLMs to generate proteins tailored for specific functions. Experimental results demonstrate the effectiveness of PrefixProt in generating proteins with desired properties,^126^ offering a flexible approach to controllable protein design.^126^ This approach demonstrates superior performance and efficiency compared to traditional fine-tuning and reduces the number of trainable parameters. Future research will focus on integrating multi-modal information for protein generation and extending these techniques to applications in antibody and drug peptide discovery, likely pushing the boundaries of drug discovery research.^126^

Generative diffusion probabilistic models, such as RF diffusion, explore vast protein landscapes while preserving backbone structures. These models can support AMP drug design by incorporating generative techniques, providing novel approaches for discovering AMP candidates.^128^

Combining pLMs with diffusion probabilistic models offers a novel generative strategy for AMP design.^128^ One example is ProT-Diff, which uses a pre-trained pLM to extract AMP features for clustering and peptide embeddings. The diffusion process reconstructs and denoises these embeddings, generating new peptide sequences^128^ (Figure 5). The diffusion protein language model (DPLM) excels in generative and predictive tasks, generating novel and structurally plausible protein sequences without conditioning by leveraging large-scale evolutionary protein data in a self-supervised framework.

This approach generalizes protein language modeling, enabling DPLMs to generate novel and structurally plausible protein sequences without conditioning.^129^ AMP-Diffusion, a latent space diffusion model tailored for AMP generation, leverages the ESM-2 protein language model (pLM) to produce functional AMPs *de novo*, facilitating their application in experimental settings.^130^ Similarly, DiMA applies continuous diffusion on embeddings generated by ESM-2, reliably producing diverse and biologically significant amino acid sequences and furthering advancements in protein design and discovery.^131^

ProteinMPNN, a graph neural network-based generative model, enables the design of novel proteins that models like AlphaFold2 or AlphaFold3 cannot yet create,^132^ including cyclic oligomers^133^ and protein-protein interfaces.^134^ By combining ProteinMPNN with LLMs for prediction, we can now address challenges in designing peptides and AMPs with desired functions. Equivariant structure prediction networks, such as OmegaFold,^135^ ESMFold,^136,137^ and RoseTTAFold,^137^ can also enhance denoising in diffusion probabilistic models. For example, RFdifussion^20^ uses RoseTTaFold as a denoising network to refine protein structures. While not exclusively focused on peptide or AMP design, models like Chroma^138^ demonstrate potential in generating proteins with specific structural or functional properties, surpassing traditional diffusion techniques.^20^ These models can be adapted for peptide and AMP design to prioritize antimicrobial activity or other desired functions, ultimately improving the generation of compounds with targeted activity or functionality.

## CHALLENGES AND OPPORTUNITIES

Integrating LLMs into antibiotic research holds promise, but significant challenges remain, such as limited data on effective antibiotics, resistance mechanisms, and the economic feasibility of drug development.^139^ However, these obstacles present opportunities for breakthroughs in discovering and optimizing new antimicrobials.

While LLMs significantly contribute to advancing protein LLMs in peptide and AMP studies, we have to acknowledge that their substantial computational resource requirements pose a challenge. As the number of parameters in LLMs increases, many biological laboratories face resource constraints, hindering using these models and developing new models based on them. Consequently, balancing performance and resource costs becomes imperative. However, few comprehensively examine the direct relationship between parameter quantity and model performance. This research direction holds significant importance, and future studies are encouraged to address this knowledge gap.

LLMs are efficient at parsing vast scientific literature and databases to uncover relationships between molecular structures and antimicrobial activity. However, molecular discovery is still in its early stages, with challenges like insufficient training data and false positives limiting the accuracy of LLM predictions. Integrating additional knowledge, such as Gene Ontology (GO), into protein LLMs could enhance model performance. Innovative data augmentation techniques can also address the issue of limited training data. To reduce false positives, combining multiple predictors or employing multimodal models could help improve accuracy. Additionally, the complexity of multidrug-resistant pathogens and the high failure rate of clinical trials due to toxicity and limited activity spectra highlight the urgent need for safer and more effective antibiotic candidates.

Innovative approaches, such as combining LLMs with high-throughput screening methods and using generative LLMs in agentic workflows, could help innovate how antibiotics are discovered, moving away from traditional, time-consuming experimental methods. As antibiotic resistance rapidly emerges, it is critical to optimize current treatments and develop new antibiotics that are less likely to induce resistance.

LLMs also offer the potential to design antibiotics that bypass resistance mechanisms and target a broader spectrum of bacteria. Property optimization in LLMs should address multiple properties simultaneously—activity, toxicity, and solubility—to accelerate the clinical development of AMPs. Reinforcement learning (RL) frameworks, like REINVENT and DeepRL, have already been applied to molecular design, optimizing multiple objectives. Adapting these RL-based approaches to antibiotic design could enable LLMs to improve candidate molecules using feedback from experiments or simulations iteratively.

In conclusion, LLMs offer a novel avenue to combat evolving pathogens. They hold potential to accelerate the discovery, design, and optimization of antibiotics and ensure their continued effectiveness in an increasingly resistant microbial landscape.

## Figures and Tables

**Table 1.** Loss functions of pre-training tasks

Task	Loss function	Description

MLM	$L_{MLM}=-\sum_{\overset{ˆ}{x}\;\in m(x)}logp\left(\hat{x}\backslash x_{\backslash m(x)}\right)$	m(x) and $x_{\backslash m(x)}$ denote the masked words from x and the remaining words, respectively
NSP	$L_{NSP}=-logp(t|x,y)$	t=1 if x and y are continuous segments from corpus
LM	$L_{LM}=-\sum_{t\;=\;1}^{T}logp\left(x_{t}|x_{\langle t<}\right)$	$x_{\langle t<}=x_{1},x_{2},\ldots ,x_{t-1}$

**Table 2.** List of representative pre-trained protein LLMs and structure models

Model	Variants	Architecture	Pre-trained task	Parameter	Layer	Application for peptide	URL

Tape	–	BERT	LM and MLM	38 M	12	peptide-MHC binding prediction, AMP prediction, peptide solubility prediction	https://github.com/songlab-cal/tape
ProtTrans	ProtAlbert	BERT	MLM	224 M	12	peptide-MHC binding prediction, AMP prediction, peptide toxicity prediction, peptide stability prediction, cleavage site prediction, signal peptide prediction	https://github.com/agemagician/ProtTrans
ProtTrans	ProtBERT	BERT	MLM	420 M	30	peptide-MHC binding prediction, AMP prediction, peptide toxicity prediction, peptide stability p rediction, cleavage site prediction, signal peptide prediction	https://github.com/agemagician/ProtTrans
ProtTrans	ProtElectra	BERT	MLM	420 M	30	peptide-MHC binding prediction, AMP prediction, peptide toxicity prediction, peptide stability prediction, cleavage site prediction, signal peptide prediction	https://github.com/agemagician/ProtTrans
ProtTrans	ProtXLNet	GPT	MLM	409 M	30	peptide-MHC binding prediction, AMP prediction, peptide toxicity prediction, peptide stability prediction, cleavage site prediction, signal peptide prediction	https://github.com/agemagician/ProtTrans
ProtTrans	ProtTXL	GPT	MLM	562 M	32	peptide-MHC binding prediction, AMP prediction, peptide toxicity prediction, peptide stability prediction, cleavage site prediction, signal peptide prediction	https://github.com/agemagician/ProtTrans
ProtTrans	ProtT5-XL	Transformer	MLM	3 B	24	peptide-MHC binding prediction, AMP prediction, peptide toxicity prediction, peptide stability prediction, cleavage site prediction, signal peptide prediction	https://github.com/agemagician/ProtTrans
ProtTrans	ProtT5-XXL	Transformer	MLM	11 B	24	peptide-MHC binding prediction, AMP prediction, peptide toxicity prediction, peptide stability prediction, cleavage site prediction, signal peptide prediction	https://github.com/agemagician/ProtTrans
ESM-1	esm1_t6_43M_UR50S	BERT	MLM	43 M	6	epitope and allergenicity prediction, peptide-MHC binding prediction, peptide toxicity prediction, peptide solubility prediction, enzymepeptide cleavage prediction	https://github.com/facebookresearch/esm
ESM-1	esm1_t12_85M_UR50S	BERT	MLM	85 M	12	epitope and allergenicity prediction, peptide-MHC binding prediction, peptide toxicity prediction, peptide solubility prediction, enzymepeptide cleavage prediction	https://github.com/facebookresearch/esm
ESM-1	esm1_t34_670M_UR100	BERT	MLM	670 M	34	epitope and allergenicity prediction, peptide-MHC binding prediction, peptide toxicity prediction, peptide solubility prediction, enzymepeptide cleavage prediction	https://github.com/facebookresearch/esm
ESM-1	esm1_t34_670M_UR50D	BERT	MLM	670 M	34	epitope and allergenicity prediction, peptide-MHC binding prediction, peptide toxicity prediction, peptide solubility prediction, enzymepeptide cleavage prediction	https://github.com/facebookresearch/esm
ESM-1	esm1_t34_670M_UR50S	BERT	MLM	670 M	34	epitope and allergenicity prediction, peptide-MHC binding prediction, peptide toxicity prediction, peptide solubility prediction, enzymepeptide cleavage prediction	https://github.com/facebookresearch/esm
ESM-1b	esm1b_t33_650M_UR50S	BERT	MLM	650 M	33	peptide-MHC binding prediction, AMP prediction, peptide stability and aggregation prediction, protein-peptide interaction prediction	https://github.com/facebookresearch/esm
ESM-MSA-1	esm_msa1_t12_100M_UR50S	BERT	MLM	100 M	12	peptide design	https://github.com/facebookresearch/esm
ESM-MSA-1b	esm_msa1b_t12_100M_UR50S	BERT	MLM	100 M	12	peptide design	https://github.com/facebookresearch/esm
ESM-2	esm2_t6_8M_UR50D	BERT	MLM	8 M	6	AMP prediction, peptide binder, anticancer peptides, cell-penetrating peptide prediction	https://github.com/facebookresearch/esm
ESM-2	esm2_t12_35M_UR50D	BERT	MLM	35 M	12	AMP prediction, peptide binder, anticancer peptides, cell- penetrating peptide prediction	https://github.com/facebookresearch/esm
ESM-2	esm2_t30_150M_UR50D	BERT	MLM	150 M	30	AMP prediction, peptide binder, anticancer peptides, cell- penetrating peptide prediction	https://github.com/facebookresearch/esm
ESM-2	esm2_t33_650M_UR50D	BERT	MLM	650 M	33	AMP prediction, peptide binder, anticancer peptides, cell- penetrating peptide prediction	https://github.com/facebookresearch/esm
ESM-2	esm2_t36_3B_UR50D	BERT	MLM	3 B	36	AMP prediction, peptide binder, anticancer peptides, cell-penetrating peptide prediction	https://github.com/facebookresearch/esm
ESM-2	esm2_t48_15B_UR50D	BERT	MLM	15 B	48	AMP prediction, peptide binder, anticancer peptides, cell-penetrating peptide prediction	https://github.com/facebookresearch/esm
ESM-3	esm3-open-small	BERT	MLM with different mask rate	1.4 B	48	–	https://github.com/evolutionaryscale/esm
ESM-3	esm3-medium	BERT	MLM with different mask rate	7 B	96	–	https://github.com/evolutionaryscale/esm
ESM-3	esm3-large	BERT	MLM with different mask rate	98 B	216	–	https://github.com/evolutionaryscale/esm
ProtGPT-2	–	GPT	LM	738 M	36	design of AMPs	https://huggingface.co/nferruz/ProtGPT2
ProGen	–	GPT	LM	1.2 B	36	–	https://zenodo.org/records/7296780
progen2	progen2-small	GPT	LM	151 M	12	antibody and peptide-specific generation	https://github.com/salesforce/progen/tree/main/progen2
progen2	progen2-medium	GPT	LM	764 M	27	antibody and peptide-specific generation	https://github.com/salesforce/progen/tree/main/progen2
progen2	progen2-oas	GPT	LM	764 M	27	antibody and peptide-specific generation	https://github.com/salesforce/progen/tree/main/progen2
progen2	progen2-base	GPT	LM	764 M	27	antibody and peptide-specific generation	https://github.com/salesforce/progen/tree/main/progen2
progen2	progen2-large	GPT	LM	2.7 B	32	antibody and peptide-specific generation	https://github.com/salesforce/progen/tree/main/progen2
progen2	progen2-xlarge	GPT	LM	6.4 B	32	antibody and peptide-specific generation	https://github.com/salesforce/progen/tree/main/progen2
ProteinBERT	–	BERT	MLM and GO prediction	16 M	8	AMP prediction	https://github.com/nadavbra/protein_bert
Tranception	–	GPT	LM	700 M	36	–	https://arxiv.org/abs/2205.13760
PRoBERTa	–	BERT	MLM	44 M	7	–	https://github.com/annambiar/PRoBERTa
PMLM	–	BERT	MLM	715 M	34	–	https://arxiv.org/abs/2110.15527
ProteinLM	–	BERT	MLM	3 B	24	–	https://github.com/THUDM/ProteinLM
AminoBERT	–	BERT	MLM	–	12	–	https://github.com/aqlaboratory/rgn2
RITA	–	GPT	LM	1.2 B	24	–	https://github.com/lightonai/RITA
xTrimoPGLM	–	BERT	MLM	100 B	30	–	https://arxiv.org/abs/2401.06199
ReprogBERT	–	BERT	MLM	110 M	12	–	https://github.com/IBM/ReprogBERT
PoET	–	GPT	LM	604 M	16	–	https://github.com/OpenProteinAI/PoET
CELL-E2	–	BERT	MLM	35 M	12	–	https://bohuanglab.github.io/CELL-E_2
ProtHyena	–	GPT	LM	1.6 M	4	–	https://www.biorxiv.org/content/10.1101/2024.01.18.576206v1
SaProt	–	BERT	MLM	650 M	33	–	https://github.com/westlake-repl/SaProt
OmegaPLM	–	BERT	MLM	670 M	66	–	https://www.biorxiv.org/content/10.1101/2022.07.21.500999v1

**Table 3.** Applications of large language models in antimicrobial peptide development

Classification	Model	Foundation LLMs	Tuning	Tasks

Property prediction	TransImbAMP	Tape	fine-tuning	AMP prediction and their functional activities prediction
Property prediction	AMP BERT	ProtBERT	fine-tuning	AMP prediction and classification
Property prediction	PeptideBert	ProtBERT	fine-tuning	AMP nonfouling/hemolysis/ solubility prediction
Property prediction	KT-AMP	ProtT5	fine-tuning	AMP prediction and other function of AMP
Property prediction	iAMP-bert	ESM-2	fine-tuning	AMP prediction
Property prediction	AMP-ProteinBERT	ProteinBERT	fine-tuning	AMP prediction
Property prediction	Bert-Protein	–	fine-tuning	AMP prediction
Property prediction	cAMPs_pred	NLP BERT	fine-tuning	AMP prediction
Property prediction	GPT-3	GPT-3	fine-tuning	AMP activity and hemolysis prediction
Property prediction	LMPred	ProtTrans	contextualized embeddings	AMP prediction
Property prediction	AMP-Detector	ProTrans, ESM1b	contextualized embeddings	AMP prediction
Property prediction	UniproLcad	ProtBERT, ESM-2	contextualized embeddings	AMP prediction
Property prediction	PepNet	ProtT5	contextualized embeddings	AMP and anti-inflammatory peptide prediction
Property prediction	SenseXAMP	ESM1b	contextualized embeddings	AMP prediction
Property prediction	iAMP-Attenpred	NLP BERT	contextualized embeddings	AMP prediction
Design	PrefixProt	ProtGPT2	prefix tuning	generating AMP and ACP with desired properties
Design	ProT-Diff	ProtT5	combining diffusion processing	AMP generation
Design	AMP-Diffusion	ESM2	combining diffusion processing	AMP generation
